# Microscopy‐guided laser ablation for the creation of complex skin models with folliculoid appendages

**DOI:** 10.1002/btm2.10195

**Published:** 2020-12-15

**Authors:** Carla M. Abreu, Luca Gasperini, Manuela E. L. Lago, Rui L. Reis, Alexandra P. Marques

**Affiliations:** ^1^ 3B's Research Group – Biomaterials, Biodegradables and Biomimetics, Headquarters of the European Institute of Excellence on Tissue Engineering and Regenerative Medicine, University of Minho Guimarães Portugal; ^2^ ICVS/3B's – PT Government Associate Laboratory Braga/Guimarães Portugal

**Keywords:** 3D tissue model, biofabrication, dermal papilla, hair follicle, laser ablation, skin model

## Abstract

Engineering complex tissues requires the use of advanced biofabrication techniques that allow the replication of the tissue's 3D microenvironment, architecture and cellular interactions. In the case of skin, the most successful strategies to introduce the complexity of hair follicle (HF) appendages have highlighted the importance of facilitating direct interaction between dermal papilla (DP) cells and keratinocytes (KCs) in organotypic skin models. In this work, we took advantage of microscopy‐guided laser ablation (MGLA) to microfabricate a fibroblast‐populated collagen hydrogel and create a subcompartment that guides the migration of KCs and lead their interaction with DP cells to recreate follicular structures. Upon definition of the processing parameters (laser incidence area and power), MGLA was used to create 3D microchannels from the surface of a standard organotypic human skin model up to the aggregates containing DP cells and KCs, previously incorporated into the dermal‐like fibroblast‐collagen layer. Analysis of the constructs showed that the fabricated microfeatures successfully guided the fusion between epidermal and aggregates keratinocytes, which differentiated into follicular‐like structures within the organotypic human skin model, increasing its functionality. In summary, we demonstrate the fabrication of a highly structured 3D hydrogel‐based construct using MGLA to attain a complex skin model bearing folliculoid structures, highlighting its potential use as an in vitro platform to study the mechanisms controlling HF development or for the screening of bioactive substances.

## INTRODUCTION

Complex tissue models that replicate human biological interactions and the tissues' 3D architecture and function are a requirement for the development of accurate and reliable in vitro systems. Recent advances in biofabrication techniques, such as 3D‐printing, micromolding, and soft lithography have contributed to the production of 3D tissue constructs with increased complexity,^1^, ^2^ but many features remain unaccomplished.

In the case of skin, currently available models are far from replicating its complexity, representing an oversimplified version composed only by the epidermal and dermal layers. Contrastingly, the skin is an organ endowed with important physiological functions, which are conferred by the presence of specialized cell populations and by functional skin appendages. The hair follicle (HF) critically contributes to the most important physiological functions attributed to skin, including barrier function, thermoregulation, sensory perception, and immunosurveillance. Consequently, the lack of HFs in skin models has hampered their translational value in the pharmaceutical and biomedical fields. In particular, given the HF high cosmetic value, there is a great deal of interest in prompting HF regenerative therapies, which cannot be dissociated from the development of appropriate test systems representative of the HF formation events, to find novel targeted treatments/drugs.

It is well established that HF development depends on reciprocal interactions between its epithelial and mesenchymal compartments (EMIs), in which the inductive dermal papilla (DP) cells stimulate the overlying epithelial cells to proliferate and differentiate into the distinct HF epithelial layers.^3^ The recreation of these processes in vitro faces some challenges, such as the loss of inductivity that DP cells suffer upon 2D culture, partially recovered in 3D spheroids,^4^ as well as the need of adequate positional and microenvironmental conditions in which EMIs can be re‐established. The most successful strategies achieved so far rely on the modification of a standard organotypic human skin model, either by incorporating DP cell‐spheroids within the dermal‐like fibroblast‐collagen layer^5^ or by creating in situ conditions that allow DP cells to self‐organize as spheroids prior the seeding of keratinocytes (KCs).^6^ While the first showed a potential communication between DP cells and epidermal KCs, which resulted in epidermal invaginations toward the spheroids but no HF‐like structures formation,^5^ the second approach confirmed the need for a direct interaction between these cells to promote follicular differentiation in organotypic skin models.^6^

Laser ablation is a noncontact technique that allows the removal of successive fractions of material by irradiation with a pulsed laser beam. In this process, the energy of the laser photons is transferred to the electrons of the target material increasing the temperature until the material vaporizes.^7^ Laser ablation techniques have a wide range of applications, which include laser surgery, selective cell ablation in basic research, patterning/modification of surfaces and the engineering of the cell microenvironment.^8^ Moreover, it enables the precise micropatterning of three‐dimensional scaffolds with high degree of control and precision over degraded features, the reason why it has emerged as a promising tool in the bioengineering field.^8^ Among others, laser‐based hydrogel degradation allows the production of scaffolds with channels capable of guiding the cellular organization and migration.^8^ For example, Sarig‐Nadir et al.^9^ ablated channels in a PEGylated fibrinogen hydrogel to direct neurites growth and create 3D neuronal networks in vitro. Ilina and coworkers^10^ created 3D microtracks in collagen matrices to support and guide breast cancer cells extracellular matrix (ECM) invasion.

Here, we report the use of microscopy‐guided laser ablation (MGLA) for the microscale manipulation of a fibroblast‐populated collagen hydrogel and the fabrication of microfeatures that enable the recreation of HFs in an organotypic human skin model. We defined the laser incidence area and power parameters to successfully ablate collagen material with minor impact in the viability of the cells already growing within it. Considering previous findings, regarding the importance of DP cells–KCs interactions,^6^ cell aggregates formed by DP cells spheroids enclosed by KCs were incorporated into the fibroblast‐collagen layer. MPGA was used to create 3D microchannels from the surface of the model up to the aggregates to guide the migration of the KCs seeded on top. Morphological analysis of the constructs demonstrated that the created MPGA microchannels successfully allowed recreating the DP and epithelial cells arrangement as observed in the HF, and the establishment of the necessary interactions to generate HF‐like structures. Overall, a skin model with follicular appendages was biofabricated using MGLA to fine‐tune both the model biological and spatial properties, ultimately increasing its level of complexity and functionality.

## RESULTS AND DISCUSSION

**FIGURE 1:**
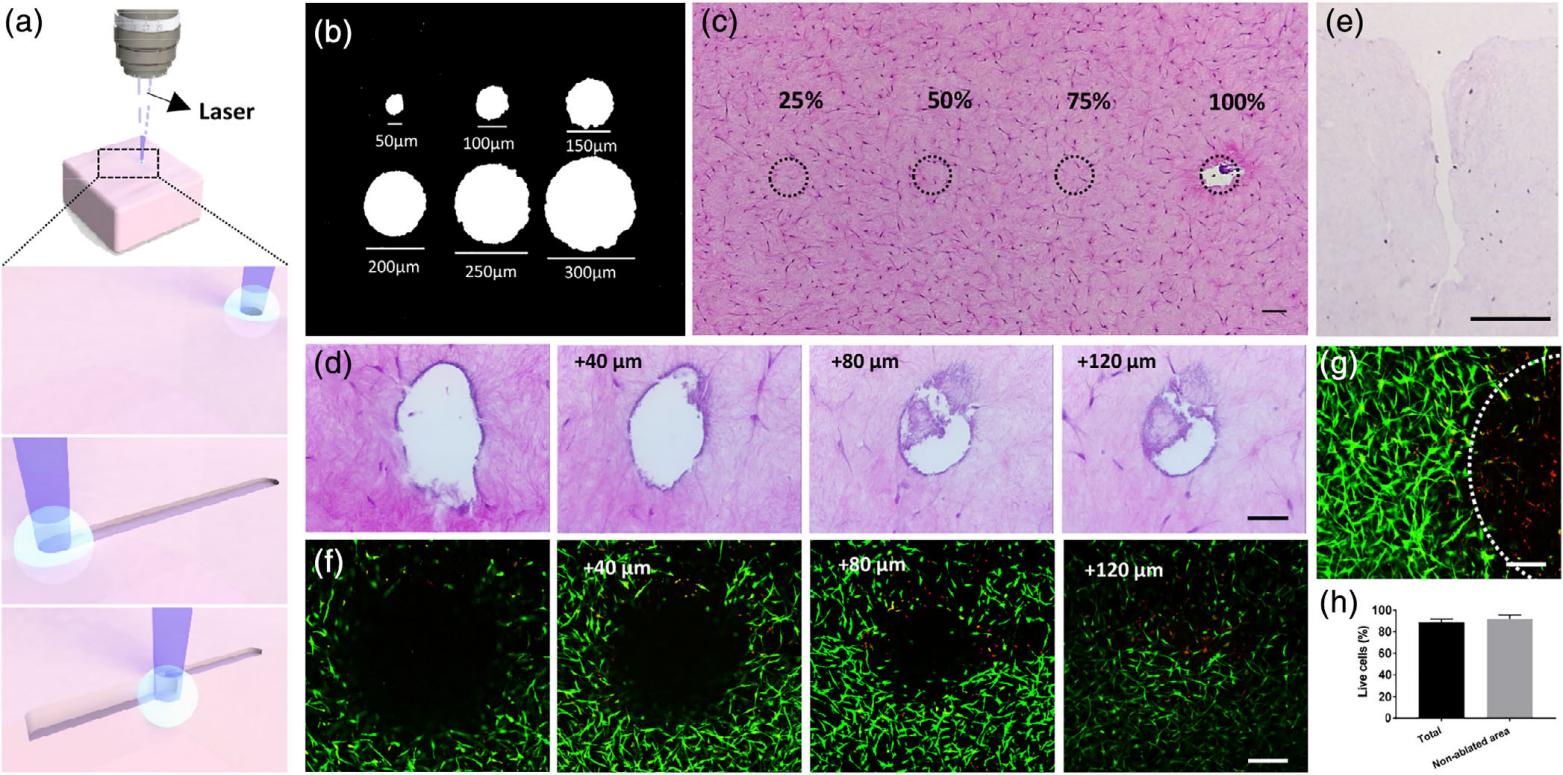
Microscopy‐guided laser ablation (MGLA) principle and conditions. (a) At the focus and delimited spot of the laser, the material is vaporized due to thermal decomposition, creating an ablation point. A continuous scan allows creating continuous microfeatures that can be deepened by changing the z‐plane of focus and repeating the ablation scanning. (b) Diameter of the holes attained in a glass slide when different ablation diameters were set in the software. (c) Top view of an H&E stained collagen cell‐laden hydrogel showing the potential ablation areas (dashed circle) using laser with different powers, demonstrating that the laser full power (100%) is required to ablate the collagen matrices. (d) H&E images of successive sections of a 300 μm depth channel done in the collagen cell‐laden hydrogel, showing the void space created by the channel along with increasing depth. (e) Transversal cut of the channel done in the collagen cell‐laden hydrogel after 7 days in culture confirming that it was kept open, despite the changes in relation to the initial dimensions. Representative images of calcein (live cells, green) and propidium iodide (dead cells, red) stained fibroblast populating the hydrogels 24 h after ablation (up to 120 μm depth) showing the presence of dead cells only in the (f) border and (g) near the bottom of the ablated area (dashed circle), and (h) the percentage of viable cells quantified considering the ablated area (total) or not (nonablated area). Scale bars are 250 μm for (f, g), 100 μm for (c, e) and 50 μm for (d)

During laser ablation, when a high‐peaked power pulsed laser is focused on a sample the material in the area affected by the laser vaporizes. The laser causes a photoinduced breaking of bonds and a thermal decomposition of the material with very little damage to the surrounding area.^11^ Thus, micro‐laser ablation allows ablating a selected portion of material delimited by the spot size of the laser. The laser can then scan a predefined area of the sample, instantaneously removing the material along the path. Once all the area is ablated, the z‐plane focus is moved to repeat the scan and ablate in depth the successive layer of material (Figure 1(a)). Laser‐based ablation has been mostly used in hard materials, and knowing that the resolution and efficiency of the process in hydrogels is both dependent on the laser characteristic and the material properties,^8^, ^12^ we first optimized the ablation parameters. To confirm the accuracy of the ablation, we first set different ablation diameters in the software—from 50 μm to 300 μm with 50 μm increments—and assessed the real diameter of the holes created in a black microscopy slide (Figure 1(b)). The results showed that the diameter of the ablated spots corresponded to the settings. Then, we proceeded with the identification of the laser power suitable to ablate our target material, fibroblast cell‐laden collagen hydrogels. We adjusted the software to ablate four separate holes (150 μm diameter) of the same sample, at different laser powers from 25% to 100%, with 25% increments. Histological analysis demonstrated that collagen ablation requires the use of the laser at full power, since other conditions did not remove any portion of the surface of the construct (Figure 1(c)). Since the diameter of the hole obtained at full power corresponds to the one set in the software, this experiment also allowed confirming the accuracy of the process in the collagen gel, as it was observed for the glass slide. After defining the working conditions, we assessed if a continuous ablation in the z‐axis would result in the successive removal of the material and formation of a continuous channel. The ablation at different depths toward the opposite side of the hydrogel surface was confirmed (Figure 1D, Supplementary video S1). The ablation of the first layer was associated with the formation of bubbles between the collagen gel and the glass slide and was also confirmed by a change in the hydrogel transparency (Supplementary video S2). The observed bubbles may be due to the effects of the laser ablation on our water‐rich samples.^13^ The channels showed the presence of some of the ablated material, which might be associated with losses in the laser beam power along the optical path, through processes such as energy diffusion or collision with heavier particles,^8^ therefore impacting the laser efficiency in deeper areas. Moreover, we also observed slight variations regarding the microchannel dimension and shape, which might be due to histological processing.^14^

Since our strategy involves the seeding of KCs on top of the ablated hydrogels and their migration and proliferation inside the void space of the channels, we assessed if the dimensions of the channel were affected by collagen contraction. In fibroblast‐laden collagen hydrogels cultured for 7 days, the size of the channel was considerably affected (Figure 1(e)), probably due to matrix contraction.^15^ The channel, initially with 150 μm in diameter and 510 μm in depth, suffered higher contraction in the deepest part widening towards the surface and ending up with 135 μm diameter at the surface and 300 μm in depth. This demonstrates that even if the matrix contracts, KCs will still be able to infiltrate the channels. A potential side effect of MGLA in the encapsulated cells was also assessed by testing cellular viability on the day after the procedure. As expected, we verified the presence of some dead cells around the ablated channel (Figure 1(f)) and in the proximity of the bottom but without impacting the viability of the surrounding cells (Figure 1(g,h), Supplementary video S3).

**FIGURE 2:**
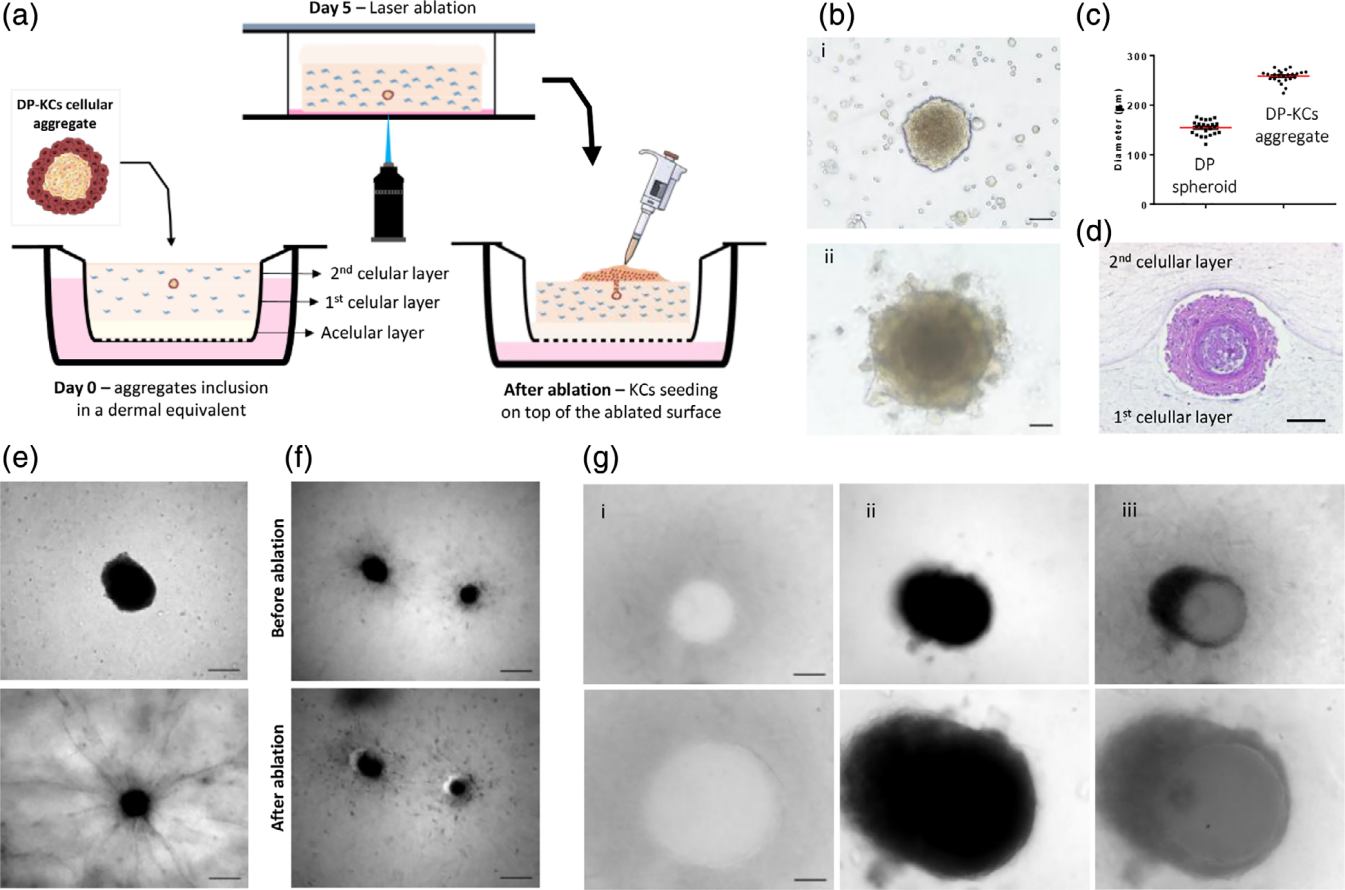
Ablation of 3D microchannels in the fibroblast‐laden hydrogels. (a) Schematic representation of the procedure adopted to incorporate DP cells‐KCs aggregates between fibroblast‐embedded collagen layers, the ablation of a microchannel connecting the hydrogel surface to the aggregate and the subsequent seeding of KCs on top of the ablated surface. (b) Phase‐contrast image of DP spheroids co‐cultured with a suspension of KCs (i) and the DP cells‐KCs aggregates formed after 2 days of culture (ii). (c) Quantification of the diameter of DP cells‐KCs aggregates and DP spheroids. (d) Representative H&E image of a DP cells‐KCs cellular aggregate 5 days after its incorporation in the dermal‐like collagen layer. (e) Representative light microscopy images of a DP cells‐KCs aggregate (top) and a DP spheroid (bottom) inside the collagen matrices after 5 days in culture, respectively showing maintenance of the structure and the outgrowth of the DP cells into the collagen. (f) Light microscopy images of DP spheroids before and after the creation of an off‐centered microchannel by MGLA. (g) Light microscopy images of the microchannel at the top of the hydrogel (i) and just before the DP cells‐KCs aggregate (ii), and the corresponding overlap (iii), denoting the position of the ablated microchannels in relation to the DP cells‐KCs aggregate. Bottom images are higher magnifications of the top ones. Scale bars are 200 μm for (e—top image, f), 100 μm for (d, g—top image) and 50 μm for (b, e, and g—bottom images)

Having determined the laser ablation parameters that allow the removal of collagen with minimal impact on cell viability, we next used MPGA to create 3D microchannels in the cell‐laden hydrogels (Figure 2(a)). DP cells spheroids were prepared and directly cultured with KCs, forming compartmentalized aggregates (Figure 2(b)) with a mean diameter of 258.5 ± 2.5 μm (Figure 2(c)), that replicated the cells 3D‐positional relationship in vivo during hair growth.^16^ These DP cells‐KCs aggregates, were then incorporated in the dermal equivalents, between the cellular collagen layers, working as hair‐forming units. The fibroblasts were let to populated the collagen, produce ECM and remodel the collagen, causing its contraction.^17^ After contraction, samples maintained transparency, which allowed the localization of the aggregates within their collagen bed. During the whole culture and contraction of the model, the overall structure and compartmentalization of the multicellular aggregates were kept (Figure 2(d); Figure 2(e), upper image). On the contrary, the diameter of the control DP spheroids without KCs decreased and DP cells outgrew into the hydrogels (Figure 2(e), lower image). While representing a specialized follicular population, DP cells can easily suffer a dedifferentiation process and successfully replace fibroblasts as the dermal cellular source in skin constructs.^18^ Therefore, the pre‐culture of DP cells with KCs, and consequent aggregate formation, is of critical importance for the generation of individual and compartmentalized HF units. During the ablation procedure, a channel with 150 μm diameter was created from the collagen surface to the cellular aggregates (Supplementary video S4). For an unequivocal demonstration that the created channels reached up to the spheroid we created channels slightly off‐centered (Figure 2(f)), however, in our constructs, the channels were aligned with the aggregate (Figure 2(g)).

**FIGURE 3:**
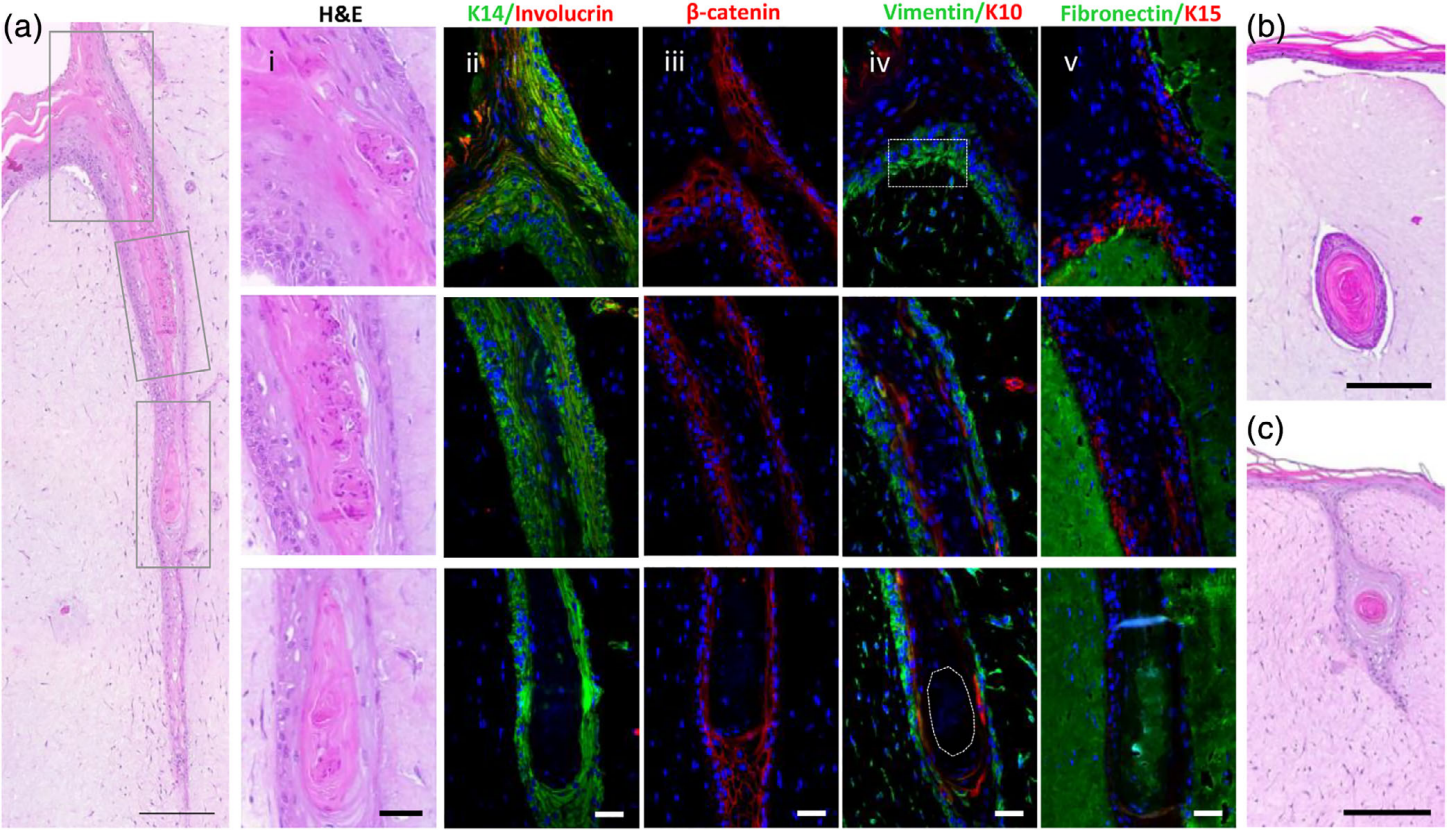
Organotypic human skin model with follicular units. (a) Representative H&E images of the HF‐like structures replicating the native tissue architecture and including (i) an epidermal invagination (top), a middle portion (middle) and a hair bulb mimetic area (bottom). Representative immunohistochemistry images showing the expression of (ii) K14 (green), involucrin (red), (iii) β‐catenin (red), (iv) vimentin (green), K10 (red), (v) fibronectin (green) and K15 (red) within the recreated follicular structures. Nuclei were counterstained with DAPI. Representative images of control organotypic human models (b) with DP cells‐KCs aggregates incorporated in the fibroblast‐seeded hydrogels but without the microchannel, and (c) with the microchannel but without the DP cells‐KCs aggregates. Scale bars are 250 μm for (a), 200 μm for (b, c) and 50 μm for (i–v)

KCs were able to infiltrate the ablated channel and form a multilayered epithelium that integrated with the DP cells‐KCs aggregates, leading to the formation of structures (Figure 3(a) i) that morphologically resembled an immature hair bulb (Figure S1(a)). The epithelium of the formed HF‐like structures exhibited a complexity by far higher than the epidermis on the dermal equivalents without the channels (Figure 3(b)). The formation of channels in hydrogels without aggregates also enabled KCs to grow downward, but the resultant epithelial strand shared the same simple morphological features of the epidermis of the standard organotypic model, with the difference that terminal differentiation was orientated inward (Figure 3(c)). The complexity of the HF‐like structures was further confirmed by the presence of a multilayered epithelium that was positive for the epithelial basal marker keratin(K)14 (Figure 3(a) ii). Interestingly, distinct morphologies were observed within K14‐positive cells. These were arranged in regimented layer(s) of cuboidal‐shaped cells located proximal to the dermal portion, which were adjacent to cells with a large cytoplasm and these, in turn, connected to flattened cells towards the core of the HF‐like structure. This last flattened layer already denoted a certain degree of differentiation, as demonstrated by the absence of staining against the epidermal basal marker β‐catenin (Figure 3(a) iii) and by the presence of the late differentiation marker K10 (Figure 3(a) iv), revealing an expression profile similar to the human HF (Figure S1(b–d)). Moreover, terminal differentiation was also demonstrated by involucrin‐positive staining (Figure 3(a) ii). Remarkably, vimentin staining revealed the presence of a dense and organized mesenchymal layer surrounding the HF‐like structure, suggesting the organization of a dermal sheath‐mimetic layer (Figure 3(a) iv). Analysis of the vimentin staining seems to indicate that the cells from this layer were originated from the dermal compartment (Figure 3(a) iv, dashed rectangle), suggesting a signaling within the environment around the follicular structures that also impact dermal fibroblasts. Interestingly, these fibroblasts are not positive for α‐smooth muscle actin (α‐SMA) as the dermal sheath cells (Figure S2), which can be indicative of an underway dedifferentiation process. This also seems to corroborate that the ablation laser is not inducing phenotypic alterations in the fibroblasts since they retain their original phenotype (vimentin positive cells) being able to respond to the environment with a specific spatial organization within the dermis and around the follicular structures. The area where the DP cells are located appears as a dense agglomerate of cells (Figure 3(a) iv, dashed circle) embedded in a fibronectin‐rich matrix (Figure 3(a) v). This basement membrane glycoprotein is prominently produced and expressed in the DP compartment (Figure S1(e)), including in DP spheroids,^19^ and believed to play an essential role in the mechanisms regulating EMIs, including enabling signal transduction.^20^, ^21^ The expression of K15, initially described as an epidermal stem cell marker^22^ present in the hair bulge, but whose presence was later also confirmed in the outermost outer root sheath layer (ORS) and in the epidermis basal layer,^23^ was equally studied. Interestingly, in our model cells positive for K15 (Figure 3(a) v) were present in a pattern that replicated the in vivo expression, in the epidermis basal layer and the most immature areas of the HF epithelium but not in the lower hair bulb (Figure S1(e)).

Hair shaft formation was not observed, either because of insufficient culture time or, most likely, given the lack of additional paracrine signals involved in hair growth, such as the ones derived from the adipose tissue.^24^ However, it is worth noting that the KCs used in this study were isolated from a hairless skin source, which require the influence of inductive DP cells to acquire a follicular fate.^4^, ^6^, ^25^ Therefore, the formation of the folliculoid structures in our model clearly demonstrates that they are influenced and respond to DP cells signals, demonstrating our strategy ability to elicit EMIs. Finally, the microenvironment where the cellular aggregates were inserted, namely the reconstructed dermis and even the DP ECM rich in fibronectin, may have also synergistically modulated EMIs^20^ and allowed the maintenance of the DP‐KCs positional relationship, therefore also contributing to the success of this strategy.

## CONCLUSION

In conclusion, we demonstrate that the controlled 3D‐microprocessing of soft hydrogels with an emergent biofabrication tool—MGLA—supported the induction of follicular structures within a reconstructed human skin. The features microfabricated in the fibroblast‐populated collagen hydrogels led the integration of KCs with DP cells and the subsequent formation of structures that morphologically resembled an immature hair bulb. The biofabricated human skin bearing follicular units opens new avenues in skin and hair research, providing not only an in vitro platform for studying the mechanisms controlling the HF early development but also a more complex and sophisticated alternative skin in vitro model for cosmetic testing and drug development.

## MATERIALS AND METHODS

### Cell isolation and culture

DP cells were manually microdissected^26^ from scalp samples of patients undergoing hair transplant surgery at Sanare Unicapilar (Porto, Portugal) after the patients' informed consent. Cells were cultured in Dulbecco's Modified Eagle Medium (DMEM, Sigma‐Aldrich) supplemented with 10% fetal bovine serum (FBS, Gibco) and 1% antibiotic and antimycotic solution (AB, Gibco). KCs were isolated from foreskin samples obtained at Hospital Narciso Ferreira (Braga, Portugal) after the patients' informed consent and isolated according to a previously described procedure for the isolation of skin cells.^27^ KCs were directly plated onto 3T3‐fibroblast feeders previously inactivated with 4 μg/ml mitomycin C (Sigma‐Aldrich)^28^ and cultured in complete FAD medium (DMEM/Ham's F12 medium [3:1 ratio] supplemented with 10% noninactivated FBS, 5 μg/mL insulin, 1.8 × 10^−4^M adenine, 0.5 μg/mL hydrocortisone, 10^10^M cholera toxin [Sigma‐Aldrich], 10 ng/mL epidermal growth factor [Peprotech], 1.8 mM CaCl_2_ [Merck], and 1% AB). Dermal fibroblasts (DFbs) were isolated from discarded skin collected from consenting patients undergoing routine abdominoplasty surgery at Hospital da Prelada (Porto, Portugal) and cultured in Eagle's Minimum Essential Medium‐alpha modification (α‐MEM, Sigma‐Aldrich) supplemented with 10% FBS and 1% AB.

Cells were maintained under standard cultures conditions (37°C, 5%CO_2_) in a humidified incubator and the medium was changed every 2–3 days. DFbs (passage 3–4), KCs (passage 1–2), and DP cells (passage 4–5) were used for the experiments.

### Multicellular aggregates formation

Spheroids were formed by seeding 3 × 10^3^ DP cells in round bottom ultra‐low attachment 96‐wells (Corning) in 50 μl of DMEM with 10% FBS for 2 days. Afterward, 7.5 × 10^3^ KCs were resuspended in 125 μl of Keratinocyte Serum‐Free Medium (KSFM, Gibco), added to the wells and further cultured for 2 days. Phase‐contrast images of DP spheroids after KCs seeding, and of the formed multicellular aggregates, were acquired with an AxioVert.A1 microscope (Zeiss, Germany). The diameter of the cellular aggregates and DP spheroids was analyzed using the ZEN 2 software (blue edition; Zeiss) and presented as mean ± standard error of the mean (s.e.m).

### Aggregates incorporation in a dermal equivalent

The organotypic human skin model was prepared in 12 mm Transwell® (0.4 μm pore, Corning) as previously described,^15^ with some modifications. Briefly, a collagen solution was prepared by mixing basal α‐MEM (10×) with 1 N NaOH and rat tail collagen I (3 mg/ml, Invitrogen) at a ratio of 10:2.5:87.5 and 250 μl were cast onto the inserts to prepare an acellular layer. This layer was then covered with 500 μl of the same collagen solution containing hDFbs at a concentration of 7.5 × 10^4^ cells/ml, forming the first cellular layer. After polymerization, one to two DP cells‐KCs aggregates were placed on top and a second cellular layer (250 μl) was slowly added. The constructs were cultured submerged in DP cells culture medium for 5 days before starting the MGLA procedure.

### Laser ablation

Laser ablation was performed with a UGA‐42 Caliburn motorized laser focus and a 355 nm, 1 KHz, 42 μJ/pulse pulsed laser (Rapp Optoelectronic, Germany) directly coupled to an Axio Observer 7 inverted microscope (Zeiss). Before ablation, the motorized laser focus was calibrated following the procedure provided by the manufacturer. Different diameters that limit the ablation area were tested to confirm the accuracy of the process. Moreover, different laser powers, other than its full power (42 μJ/pulse, 1 KHz), were tested to determine the minimum necessary power to successfully ablate collagen‐based hydrogels.

The dermal constructs were turned upside down in a sterile three‐well chamber microscopy glass slide (Ibidi, Germany) with culture medium and sealed under sterile conditions. For each construct, the end ablation plan (x, y, and z position) was fixed by focusing the center of the cellular aggregate (20× magnification). Then, the focus plan was moved to the surface of the construct in contact with the glass slide. To determine the starting ablation z‐plane we first focused the microscope on the glass slide and ablated a small spot, easily confirmed by the presence of corrugations and the change in the transparency of the ablated surface. The focus plan was then moved 5 μm away to ablate another area. This procedure was repeated until no sign of ablated glass was seen, thus establishing the starting plan for the ablation of the hydrogel. The ablation of 150 μm diameter sections was repeated along different planes towards the cellular aggregate and up to the end ablation plan. Each ablation removed 30 μm of material, in depth.

After ablation, the constructs were placed back in the inserts and 5 × 10^4^ KCs were seeded on top in 30 μl of KSFM. Constructs were then cultured in KSFM from the top and in DP cells medium from bellow for 1 week, to allow KCs proliferation. Afterward, they were air‐lifted and cultured in complete FAD medium to promote KCs differentiation and epidermis stratification.^15^ The medium was changed every 1–2 days for 2 weeks, after which the samples were harvested and processed for histology analysis.

### Viability assay

One day after the ablation, dermal constructs were incubated with 1 μg/ml calcein‐AM and 2 μg/ml propidium iodide (Molecular Probes) for 1 h at 37°C. Samples were observed, and images were acquired with an Olympus Fluoview FV1000 laser confocal microscope (Olympus, Japan). Image analysis for cell viability was performed using the CellProfiler™ software.^29^ In brief, the maximum 2D projection of all the image stacks was thresholded using the most satisfactory method: Otsu for PI and Robust Background for calcein.^30^ Dead and living cells were counted and the percentage of live cells was expressed as the ratio of living cells per the total number of cells in the total area or in nonablated areas around the microchannels. Results were presented as mean and s.e.m (*n* = 3).

### Histological analysis

Histological (H&E) and immunocytochemistry stainings were performed in 5 μm paraffin‐embedded sections according to routine protocols. For immunodetection, sections were deparaffinized and heat‐mediated antigen retrieval was performed using citrate buffer (pH = 6.0). The sections were then permeabilized with 0.2%Triton X‐100 for 15 min at room temperature (RT) and blocked with 3% bovine serum albumin (Sigma‐Aldrich) for 45 min a RT. The incubation with the primary antibodies against vimentin (1:50; ab92547, Abcam), keratin (K) 10 (1:100; ab9026, Abcam), K14 (1:800; PRB‐155P, BioLegend), Involucrin (1:25, ab68, Abcam), fibronectin (1:100, ab2413, Abcam), K15 (1:50, ab80522, Abcam), β‐catenin (1:100, 610,154, BD Transduction Laboratories) and α‐SMA (1:200, ab7817, Abcam) was carried out overnight at 4°C. Next day, sections were incubated with Alexa Fluor 488/594‐conjugated secondary antibodies (1:500, Molecular Probes) for 1 h at RT. Nuclei were counterstained with 4',6‐diamidino‐2‐phenylindole (DAPI) (0.02 mg/ml, Biotium) for 15 min at RT. H&E staining illustrative images were taken with a DM750 microscope (Leica, Germany) whereas immunofluorescent images were acquired with an AxioVert.A1 microscope (Zeiss).

## CONFLICT OF INTERESTS

The authors declare that there is no conflict of interest.

## AUTHOR CONTRIBUTIONS

Carla Abreu performed the experiments, analyzed the data and wrote the manuscript. Luca Gasperini performed the MGLA experiments, analyzed the data and reviewed the manuscript. Manuela Lago supported the cell culture experiments and performed the live/dead staining. Rui Reis provided general technical resources, and reviewed the manuscript. Alexandra P. Marques raised financial support and administrated the project, supervised the experiments, analyzed the data and reviewed and approved the final manuscript.

## Supporting information


**Appendix** S1: Supporting Information

Video S1

Video S2

Video S3

Video S4
