# Supplementary material for: Microscopy‐guided laser ablation for the creation of complex skin models with folliculoid appendages
Source: Bioeng Transl Med. 2020 Dec 15;6(2):e10195. doi: 10.1002/btm2.10195 (PMC8126819; doi:10.1002/btm2.10195)
Supplement: Supplementary file 1 — Appendix S1: Supporting Information [file BTM2-6-e10195-s005.DOCX]

**SUPPLEMENTARY INFORMATION**

**Microscopy-guided laser ablation for the creation of complex skin models with folliculoid appendages**

Carla M. Abreu^1,2^, Luca Gaperini^1,2^, Manuela E. L. Lago^1,2^, Rui L. Reis^1,2^, Alexandra P. Marques^1,2 *^

^1^ 3B’s Research Group – Biomaterials, Biodegradables and Biomimetics, Headquarters of the European Institute of Excellence on Tissue Engineering and Regenerative Medicine, University of Minho, Avepark 4805-017 Barco, Guimarães, Portugal;

^2^ ICVS/3B’s – PT Government Associate Laboratory, Braga/Guimarães, Portugal;

***Corresponding Author:** Alexandra Pinto Marques

^
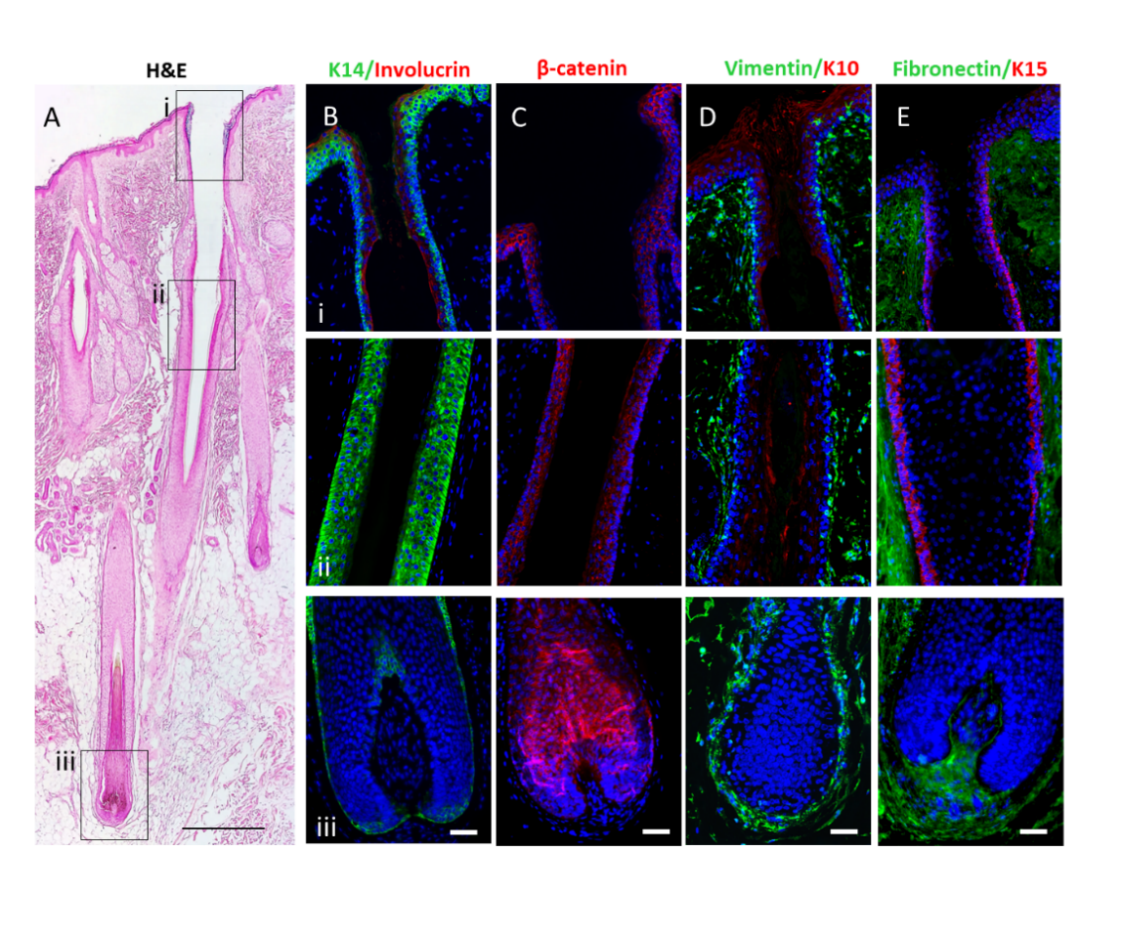
^

**Figure S1.** A) H&E images of scalp human HFs demarking the (i) epidermal invagination area, (ii) an upper middle portion and (iii) the hair bulb. Representative immunohistochemistry images showing the expression of (B) K14 (green), involucrin (red), (C) β-catenin (red), (D) vimentin (green), K10 (red), (E) fibronectin (green) and K15 (red) within the HF and surrounding microenvironment. Nuclei were counterstained with DAPI. Scale bars are 500µm for (A) and 50µm for (B-E).

**
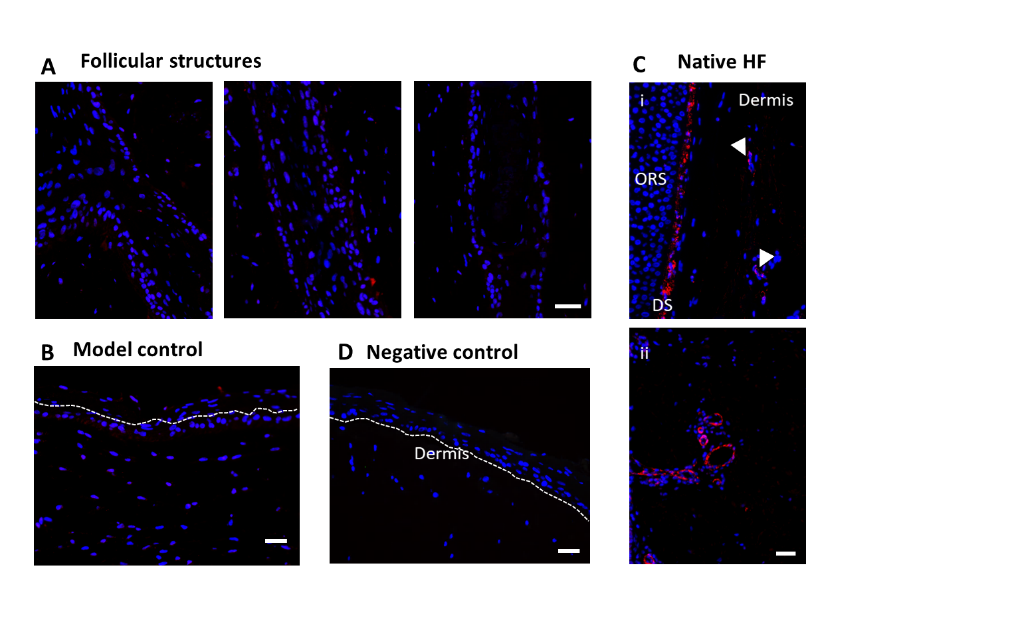
**

**Figure S2**. Figure S2. Representative immunocytochemistry images of the expression of α-SMA (red) in (A) the recreated HF-like structures and adjacent fibroblasts, and in (B) a control organotypic skin model without the DP-KCs aggregates and without ablation. (C) In the native human skin, α-SMA (red) expression can be observed in the HF dermal sheath (i) and in blood vessels present within the dermis (i- arrowhead, ii). (D) Represents the negative control for the immunostaining. Nuclei were counterstained with DAPI (blue). Scale bar = 50 µm. ORS – Outer root sheath
